# The role of ketogenic diets in the therapeutic management of adult and paediatric gliomas: a systematic review

**DOI:** 10.2217/cns-2017-0030

**Published:** 2018-04-16

**Authors:** Kirsty J Martin-McGill, Nisaharan Srikandarajah, Anthony G Marson, Catrin Tudur Smith, Michael D Jenkinson

**Affiliations:** 1 Institute of Translational Medicine, University of Liverpool & The Walton Center NHS Foundation Trust, Lower Lane, Liverpool L9 7LJ, UK; 2 Department of Biostatistics, University of Liverpool, Brownlow Hill, Liverpool L69 3BX, UK

**Keywords:** glioblastoma, glioma, ketogenic diet, systematic review

## Abstract

**Aim:**

We performed a systematic review of the evidence for effectiveness and acceptability of different ketogenic diets (KDs) in the therapeutic management of gliomas.

**Methods:**

The search strategy included searches of seven electronic databases. Data extraction and quality assessment were undertaken independently by two authors.

**Results:**

No randomized clinical trials were identified. Six studies (n = 39) met the eligibility criteria for this review – all were case series or reports and therefore at high risk of bias. All studies reported overall or progression-free survival; however the effectiveness of KD interventions could not be established. Dietary acceptability was not reported.

**Conclusion:**

The effectiveness and acceptability of KDs in the management of gliomas is unknown and high quality randomized controlled trials are needed.

**Summary points**

- This article systematically reviews the evidence for the effectiveness and acceptability of different ketogenic diets (KD) in the therapeutic management of patients with gliomas.
- Six studies have been published since 1995, conducted in the USA and Europe (n = 39).
- All studies are case series evidence, the lowest position in the evidence hierarchy. However, at present this is the only evidence available to inform decisions regarding the implementation of KDs for gliomas.
- Minimal adverse events were reported, suggesting KDs to be safe in this population.
- The evidence for effectiveness and acceptability of various KDs is insufficient to suggest they have a therapeutic effect in the management of gliomas; therefore further high-quality research is needed.
- Key areas for future research include:
  - A pragmatic feasibility study to inform future RCT design.
  - High quality randomized control trials to determine if KDs are effective in the management of glioma.
  - A health economic assessment to establish efficiency, clinical effectiveness and value of the intervention.

## Description of the condition

Primary brain tumors affect 7.14 per 100,000 of the worldwide population each year [1], with gliomas being the commonest form of malignant brain tumor, affecting three to five people per 100,000 each year [2]. Despite current treatment options including surgical resection, radiotherapy and chemotherapy, these tumors remain incurable and the prognosis is poor.

### Description of the intervention

Warburg first recognized that tumor cells rely on glucose for energy in 1926 [3]. Over the decades the Warburg theory has been developed further, leading to the hypothesis that switching gliomas’ energy source from glucose to ketones may result in cancer cell death [4]. One mechanism for achieving this is by targeting tumor energy metabolism with the ketogenic diet (KD); a high fat, low carbohydrate diet, which results in the production of ketones as a primary energy source, while minimizing glycolysis through glucose restriction [5].

KDs are perceived as unpalatable and their assessment in patients with poor survival outcomes has therefore been limited. However, following the success of the classic 4:1 KD (4 g fat for 1 g of carbohydrate and protein combined, ∼90% total energy from fat) in pediatric epilepsy, dietary variants have been developed that are designed to be more palatable and easier to implement, with fewer side effects [6].

### Why this review is important?

A systematic review and meta-analysis of the antitumor effects of KD in mice demonstrated a prolonged survival for the KD groups compared with standard diet (mean survival time ratio = 0.85 [95% highest density interval = 0.73, 0.97]; hazard ratio = 0.55 [95% highest posterior density interval = 0.26, 0.87]) [7]. To our knowledge, no such review exists for human studies and a review of the best current evidence is required to inform decisions about service provision and the design of future clinical trials.

#### Research question & aim

Aim: to review the evidence for effectiveness and acceptability of different KDs in the therapeutic management of patients with gliomas.

## Methods

The protocol for this systematic review was registered with PROSPERO (identification number: CRD42017056752).

The population, intervention, comparison and outcomes (PICO) table below illustrates the review question and inclusion criterion (population, intervention, outcomes, setting and study design). As all study designs were considered, a comparator arm was not essential (see Table 1).

**Table 1.** **A PICO table illustrating the systematic review question and criterion.**

Review question	Is there a role for KDs in the therapeutic management of adult and paediatric gliomas?	

Population	Adults and children with glioma tumors following a KD	

Intervention	Any form of KD, with KD defined as a diet that is designed to produce ketones	

Outcomes	Objective or self-reported measures are acceptable for the following outcomes: Primary outcomes: • Overall survival • Progression-free survival	Secondary outcomes: • Adverse events • Retention rates • Quality of life • Acceptability • Tolerability • Compliance • Duration of KD • Time of dietary commencement (in relation to treatment pathway) • Ketone levels • Glucose levels

Setting	Primary, secondary or tertiary healthcare. Inpatient, outpatient or community settings	

Study design	All	

KD: Ketogenic diet.

No restriction was placed on year of study or publication status. The search was limited to English language publications.

### Search strategy

A four-part search strategy was implemented to identify suitable studies.

#### Electronic searches

The following electronic databases were searched.

- EMBASE
- PubMed
- Cochrane Library
- CINAHL Plus
- MEDLINE
- SCOPUS
- Web of Science

The primary search was undertaken on 25 January 2017, with updates identified until 18 August 2017 (see the Supplementary Appendix for an example search strategy).

#### Hand searches

References of the included studies were hand searched to identify other possible studies.

#### Study registries

The following study registries were searched:

- ClinicalTrials.gov
- The World Health Organisation International Clinical Trials Registry Platform
- UK Clinical Trials Gateway
- International Standard Randomized Controlled Trial Number Register (ISRCTN)
- National Institute of Health Clinical Trials Registry
- National Research Register Projects Database Achieve
- PROSPERO

The search was undertaken on 21 March 2017, with updates identified until 18th August 2017.

#### Other resources

Conference abstracts and posters were included in the search to identify recent studies undertaken that may not be published or are ongoing.

#### Screening of included studies

Duplicate references were removed from the search results. Prepiloted inclusion criteria were applied to titles and abstracts identified in the search results. Full text was obtained for studies identified for potential inclusion and inclusion criteria were reapplied. Following this, an expert in the field (AC Scheck) was also contacted to identify any studies that were shortly due for completion or publication.

#### Reporting results of searches

The Preferred Reporting Items for Systematic Reviews and Meta Analyses (PRIMSA) flow diagram was adopted to document the number of references located from databases and other sources, the number of duplicates removed, records screened, records excluded, full text articles screened, full text articles excluded with reasons and the number of studies included in the final review [8].

### Data extraction & quality assessment

Two authors (KJ Martin-McGill and N Srikandarajah) independently extracted data using a prepiloted data extraction form. Any discrepancies were discussed between the two authors in the first instance. A third author (MD Jenkinson) was available for consultation if disagreements would have occurred.

Although all study types were permitted for inclusion in the review, only case studies and case series were identified. Therefore the Institute of Health Economics (Canada) Case Series Quality Appraisal Checklist was selected as the appropriate quality assessment tool [9]. Again, the tool was applied by KJ Martin-McGill and N Srikandarajah.

## Results

### Description of studies

#### Results of the search

**Figure 1.**
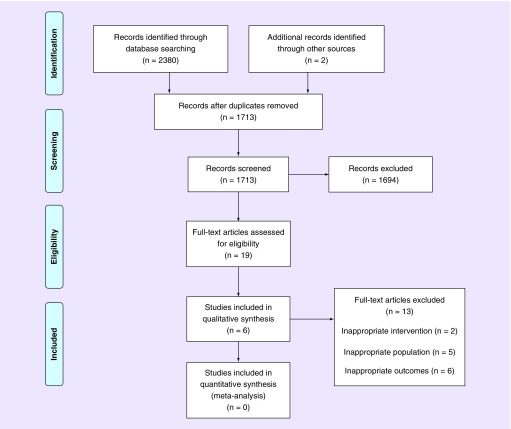
**PRISMA flow diagram.** Adapted with permission from [8] Moher *et al*. (2009).

The electronic search identified 2380 records, and another two were identified by searching the references of included studies. After removing duplicates, 1713 records remained. Following the screening of titles and abstracts, 19 remained eligible for inclusion. These studies underwent a full text review, following which a further 13 studies were excluded due to inappropriate interventions [10,11], inappropriate populations [7,12–15] and inappropriate outcome measures [16–20]. Therefore six studies met the eligibility criteria for this review (Figure 1) [21–26]. No further studies were identified from the expert in the field.

#### Included studies

Six studies have been published since 1995, conducted in the USA and Europe. Population sample size varied between one and 20 participants, with a variety of KD interventions. Table 2 provides a summary of the study characteristics.

**Table 2.** **Study characteristics.**

**Study (year)**	**Location**	**Methods**	**Primary objective**	**Number of patients**	**Age (years)**	**Diagnosis**	**Dietary intervention**	**Dietary duration (months, [range])**	**Follow-up (months, [range])**	**Survival data (median [range])**	

										**Overall survival**	**Progression-free survival**
Champ *et al*. (2014)	USA	Retrospective case series	Safety	n = 6	34–62	Grade III–IV glioma	50 g CHO KD	3–12	5–20.3	27–88 weeks (range only)	45 weeks^†^

Nebeling *et al*. (1995)	USA	Prospective case series	Nutritional status, tumor metabolism and QoL	n = 2	3–8.5	Grade IV anaplastic astrocytoma spinal cord; grade III cerebellar astrocytoma	MCT KD	2–14	2–24	0 death reported	0 progression reported

Rieger *et al*. (2014)	Germany	Prospective case series	Safety and tolerance	n = 20	30–72	GB	60 g CHO KD, fermented yoghurt drinks, two plant oils	6–42	1.2 (median only)	32 weeks (6–86+ weeks)	5 weeks (3–13 weeks)

Schwartz *et al*. (2015)	USA	Prospective case series	Unclear	n = 2	52–55	GB	ER 3:1 KD	1–3	1–3	No data	8 weeks (4–2 weeks)

Strowd *et al*. (2015)	USA	Retrospective case series	Safety and clinical impact	n = 8	28–54	Grade II–IV glioma	15–20 g CHO MAD	2–24	13.2 ± 8 (mean, SD)	0 deaths reported	No data

Zuccoli *et al*. (2010)	Italy	Retrospective case report	Unclear	n = 1	65	GB	ER 4:1 KD	1.8	25	No data	43 weeks

^†^Statistical basis uncertain from publication, unknown if data represents mean or median.

CHO: Carbohydrate; ER: Energy restriction; GB: Glioblastoma; KD: Ketogenic diet; MAD: Modified Atkins diet; MCT: Medium-chain triglyceride; QoL: Quality of life; SD: Standard deviation.

### Risk of bias in included studies

All six studies were at high risk of bias and a summary of the quality assessment can be found in Table 3.

**Table 3.** **Summary of quality assessment of included studies.**

**Study (year)**	**Objectives**	**Design**			**Population**			**Interventions and co interventions**		**Outcome measure**				**Statistical analysis**	**Results and conclusions**					**Competing interests/sources of support**

	**Aim/objectives**	**Prospective**	**Multicenter**	**Consecutive recruitment**	**Participant characteristics**	**Eligibility criteria**	**Similar point of trial entry**	**Clear intervention**	**Clear additional interventions**	**OM established priori**	**Blinded assessors**	**Appropriate methods**	**OM pre and post intervention**	**Appropriate tests**	**Follow-up period**	**Losses reported**	**Estimates of random variability**	**AE reported**	**Supported conclusions**	**Reported upon**
Champ *et al*. (2014)	✓	✗	U	✓	✓	✓	✓	✓	✓	✓✗	U	✓	U	U	✓	✓	✓✗	✓	✓	✓

Nebeling *et al*. (1995)	✓	✓	✗	U	✓	✗	✗	✓	✓	✓	U	✓✗	U	U	✓	✓	✗	✓	✓	✓

Rieger *et al*. (2014)	✓	✓	✗	✓	✓	✓	✓	✓	✓	✓	U	✓✗	U	✓	✓	✓	✓	✓	✓	✓

Schwartz *et al*. (2015)	✓✗	✓	✗	U	✓	✓	✓	✓	✓	✓✗	U	✓	✓	U	✓	✓	✗	✓	✓	✓

Strowd *et al*. (2015)	✓	✗	✗	✓	✓	✓✗	✓	✓	✓	✗	U	✗	U	✓	✓	✓	✓	✓	✓	✓

Zuccoli *et al*. (2010)	✗	✗	✗	U	✓	✗	U	✓	✓	✗	U	✓✗	U	NA	✓	✓	✗	✓	✓	✓

✓ = Yes (item adequately addressed).

✗ = No (item not adequately addressed).

✓✗ = Partial (item partially addressed).

AE: Adverse events; OM: Outcome measure; NA: Not applicable; U: Unknown.

### Effects of interventions

#### Critical outcomes

##### Overall survival

Four studies reported overall survival (OS; n = 36). Follow-up ranged from 6 to 91 weeks. Two studies comprising ten patients reported no deaths [23,26]. Two studies comprising of 22 patients reported survival ranging from 6 to 88+ weeks [21,22]. Survival can be related to diagnosis and dietary intervention in Table 2.

##### Progression-free survival

Five studies reported progression-free survival (PFS; n = 30). Time to progression ranged from 3 to 45 weeks in four studies [21–22,24–25]. One study comprising of two patients reported no progression following 62 weeks on diet, at the time of writing (n = 1); however, PFS for the second patient was not reported [26]. PFS can be related to diagnosis and dietary intervention in Table 2.

#### Important outcomes

##### Adverse events

All studies reported adverse events (n = 39). The most frequently reported adverse effects related to KD interventions were weight loss [21–25], ranging from -2.2% body weight [22] to -13% body weight [25] and increased cholesterol [24,26]. Other adverse effects reported in low numbers were deep vein thrombosis [21], grade III leukopenia [22], lymphopenia [25], hyperuricemia [25], hypoproteinemia [26].

##### Dietary retention rates

The retention rate could be determined for three studies (n = 24), all undertaken prospectively using a defined protocol, which ranged from 50 to 100% [22,24,26]. Retention was determined at 8 weeks (n = 2) [26], 12 weeks (n = 2) [24] or at the point of tumor progression (n = 20) [22] (median PFS 5 weeks, range 3–13 weeks). Reasons for withdrawal from diet included tumor progression (n = 1) [24] and negative impact on quality of life (n = 3); however no validated tool was documented [22].

##### Quality of life

No studies reported quality of life using appropriate objective or subjective measures.

##### Acceptability

No studies reported dietary acceptability using appropriate objective or subjective measures.

##### Tolerability

Two studies reported dietary tolerability (n = 18). Grade I constipation was reported at dietary initiation (n = 2), grade I fatigue (n = 4) during radiotherapy and grade II fatigue (n = 1) during 30% energy restricted 30–50 g carbohydrate KD [21]. Gastrointestinal assessment reported diarrhoea at a mean intensity of <1 (weak), constipation at a mean intensity of <1 (weak), hunger of mean intensity of >1 but <2 (weak to moderate) and demand for glucose mean intensity of >1 but <2 (weak to moderate), using a nonvalidated questionnaire (n = 12) [22].

##### Compliance

Three studies reported dietary compliance (n = 24). Maintenance of ketosis was used as a surrogate for compliance in two studies [24,26], while patient self-reporting demonstrated compliance for 6.8 days per week (n = 20) [22].

##### Ketone levels

Five studies reported ketosis (n = 24). Three studies reported serum ketosis (n = 6), with levels between levels of 0.3 mmol/l (indicates reported units have been converted to mmol/l from mg/dl for comparison) to 7 mmol/l (n = 4) [21,24] and maintenance of serum ketosis was reported by one study (n = 2) [26]. Urinary ketosis was reported by two studies (n = 14). One study reported urinary ketones between 1.5 and 2.5 mmol/l during the first 3 weeks of diet (n = 1) [25]. In the other study, urinary ketosis achieved at least once in 92% participants (n = 12/13) and when assessing all urinary measurements from 12 participants, ketonuria was present in 73% of cases [22]. Methodology and frequency of testing was not consistent between studies.

##### Glucose levels

Five studies reported serum glucose levels (n = 18). Three studies (n = 14) reported a decrease in serum glucose during diet compared with prediet levels. However, levels varied from a mean nonfasting serum glucose of 7.9 mmol/l (indicates reported units have been converted to mmol/l from mg/dl for comparison) prediet (no SD) to 4.7 mmol/l (indicates reported units have been converted to mmol/l from mg/dl for comparison) (no SD; n = 4) [21], to 7.5 mmol/l prediet decreasing to 3.5 mmol/l during diet (n = 1) [25], with a less extreme response noted in one study (5.5 ± 1.2 mmol/l [indicates reported units have been converted to mmol/l from mg/dl for comparison] prediet to 5.1 ± 0.5 mmol/l [indicates reported units have been converted to mmol/l from mg/dl for comparison] during diet; n = 9) [22]. One study reported serum glucose levels during diet only (3.5–5.5 mmol/l; n = 2) [26] and one study reported serum glucose could not be maintained below the target of 4.4 mmol/l [indicates reported units have been converted to mmol/l from mg/dl for comparison] (n = 2) [24].

### Ongoing trials

#### Results of search

Eighteen records of ongoing trials were identified within study registries. After removing duplicates, 12 records relating to 12 individual trials remained (total participant population of n = 265), all of which were eligible for this review [27–38]. Table 4 summarizes characteristics of the 12 ongoing clinical trials.

**Table 4.** **Summary of ongoing clinical trials.**

**Study (year)**	**Location**	**Population condition**	**Target sample size**	**Dietary intervention(s)**	**Primary outcomes**	**Secondary outcomes**	**Expected date of completion**
Ghodsi (2012)	Iran	Post Sx, CRT GB	20	ER MCT KD (50% MCT, ER to 25 kcal/kg/day)	Survival	Quality of life	Unknown

Guimaraes Santos & Pereira da Fonseca (2016)	Brazil	Recurrent GB	30	KD vs control with intranasal administration of perillyl alcohol	Tumor size	Anthropometry	Unknown

Jameson (2014)	New Zealand	Newly diagnosed GB	20	KD (<30 g CHO/day)	PFS	Ketosis treatment compliance Dietary compliance Food satisfaction SGA Adverse events	Unknown

Martin & Jenkinson (2017)	UK	Newly diagnosed GB	12	MKD (5% CHO, 80% fat) vs MCT KD (10% CHO, 75% fat [30% MCT])	Retention	Enrolment Long term retention Dietary adjustments Self-reported compliance Calculated compliance MCT compliance Ketosis Dietetic time Protocol refinements Sample size estimations Quality of life Food acceptability Gastrointestinal side effects Biomarkers Anthropometry Completeness of data	March 2019

Klein (2014)	USA	Recurrent GB	6	ER 4:1 KD (1600 kcals/day) vs standard diet	Overall survival PFS Adverse events	Tolerability	August 2017

Klein (2016)	USA	Newly diagnosed GB	6	ER 4:1 KD (1600 kcals/day)	Safety	Efficacy Tolerability	May 2017

Rieger & Steinbach (2012)	Germany	Recurrent GB	50	ER KD with IF (<60 g CHO/day) vs standard diet	PFS	Feasibility Safety Tolerability Overall survival Seizure frequency Ketosis Quality of life Depression Attention Response	October 2017

Scheck (2014)	USA	Newly diagnosed GB	14	4:1 KD reduced to MAD post CRT	Adverse events	Overall survival PFS Quality of life	March 2017 (study ongoing, not recruiting)

Schwartz (2012)	USA	Newly diagnosed GB	12	ER KD (20–25 kcals/kg/day)	Tumor size	None stated	July 2017

Song (2016)	China	Recurrent GB	60	KD vs standard diet	Adverse events	Chemotherapy sensitivity Overall survival Ketosis Quality of life	December 2018

Strowd (2014)	USA	Post Sx, CRT GB	25	MAD with IF	Feasibility	Tolerability Biological activity Glucose levels Ketosis Anthropometry Seizure frequency	November 2018

Vaisman (2010)	Israel	Recurrent GB	40	KD vs standard diet	Tumor size	Performance scale Quality of life	March 2011 (last updated March 2010)

CHO: Carbohydrate; CRT: Chemoradiotherapy; ER: Energy restriction; GB: Glioblastoma; IF: Intermittent fasting; KD: Ketogenic diet; MAD: Modified Atkins diet; MCT: Medium-chain triglyceride; MKD: Modified ketogenic diet; PFS: Progression-free survival; SD: Standard deviation; SGA: Subjective global assessment; Sx: Surgery.

## Discussion

### Summary of main results

This systematic review identified no high quality prospective studies assessing KD for glioma, but did identify a number of small randomized controlled trials that are currently ongoing. All six published case series included in this review reported overall or PFS; however due to the limited sample sizes (ranging from one to twenty participants) and the absence of a control group, it is not possible to make any conclusion as to the effectiveness of the KD interventions.

Adverse events were consistent across the majority of studies, predominately being weight loss and raised cholesterol. However, two studies adopted an energy restricted KD, following which weight loss would be expected [24,25]. The significance and clinical impact of weight loss would need to be considered and could be managed through nonenergy restricted, nonfasting regimes supported by a trained dietitian [26]. The impact of KD on cholesterol profiles should also be considered within the context of a disease that has poor long-term survival. While two studies reported an increase in cholesterol [24,26], one study, conflictingly reported cholesterol to reduce over the course of the diet [22]; therefore requires further investigation.

Retention rates on diet varied from 50 to 100%, however only three studies utilized a study protocol with predetermined duration for the dietary intervention [22,24,26]. As sample sizes of these studies range from 2 to 20 participants, the external validity of such data is questionable. No studies reported quality of life or dietary acceptability using the appropriate objective or subjective measures and are therefore subject to performance bias. Future studies should consider the inclusion of validated measures to assess quality of life and dietary acceptability.

Dietary compliance was inconsistently measured, with two studies citing the presence of serum ketones as a marker of compliance [24,26], and one study using self-reported measures [22]. Both methods have their limitations; including selection bias with eligibility criteria requiring patients to be compliant with the diet prior to recruitment [24] and reporter bias from self-reported measures [22]. Due to diverse methodologies, it is not possible to determine which diet is easier for participants to comply with.

A trend for the decrease in serum glucose levels, while adhering to a KD, can be noted across the studies, with glucocorticoids having a negative impact on levels. However, the clinical impact of this cannot be determined from the results of the studies so far. Five studies measured ketones in either urine or serum; however due to different methods no comparisons can be made between diets.

This review also identified 12 ongoing studies, five of which are randomized control trials (RCT). Three RCTs may be suitable for future meta-analysis [28–29,31]. These studies have comparable populations, outcome measures, control groups and similar dietary interventions, but further dietary and methodological details would be required to assess the appropriateness of such an analysis. The planned recruitment figures remain small and the trials are underpowered to demonstrate effectiveness. A multicenter RCT or the ability to undertake a meta-analysis is required.

### Potential biases in the review process

Thorough database searches were performed to identify studies suitable for this review. Application of the eligibility criteria to the research results identified six studies for inclusion. Given the relative novelty of KD in gliomas, a low number of editorials were expected. As the search strategy was first piloted and the results of the search strategy supplemented by hand searches, it is unlikely that relevant studies were missed, further confirmed through contact with an expert in the field. Therefore conclusions drawn from the review are based upon all available evidence.

A key strength of this review lies in the quality assessment of included studies. The Institute of Health Economics (IHE; Canada) Case Series Quality Appraisal Checklist [9] is the only validated quality appraisal tool for assessing the methodological quality of case series [9,39]. The tool was updated to include assessor annotations specific to this review as recommended by the authors of the tool [9], to aid the quality appraisal process. The tool does not provide a scoring system in which study quality may be distinguished as high or low, therefore it was not possible to strictly assess the confidence of cumulative evidence. As such, a narrative approach was taken in this review. No studies fulfilled the full study design criteria of the quality assessment tool.

Three studies utilized a study protocol enabling the repetition of their methods [22,24,26]. Of these, one study author [24] provided the trial protocol and there appeared to be no suggestion of selective reporting bias.

One study included a retrospective control group [21], however failed to statistically or descriptively compare the control group to the dietary intervention group. The control group was also unlikely to represent the population due to convenience sampling methods and eligibility criteria requiring variables not held within the records, thus creating selection bias. Therefore, this study design was also considered a case series.

A meta-analysis was not undertaken due to the heterogeneity of the study populations, methods, outcomes and bias stated previous. The overall measure of treatment effect would be misleading given studies were not powered to determine treatment effect. Study populations varied from newly diagnosed grade III or IV gliomas to recurrent gliomas and the KD was administered at different time points in the treatment pathway. Participants also received a wide variety of oncology treatments while following KD. The KD interventions also varied, in terms of energy and carbohydrate restrictions, and types of dietary fats included. One study included fermented yoghurts and plant oils in addition to KD [22], therefore presenting difficulties when comparing KD outcomes. Ongoing studies may provide better quality data and synergy between protocols to allow for meta-analysis in future reviews.

### Implications for practice & research

Due to the lack of high quality evidence it is difficult to justify the use of KDs in a clinical, nonresearch setting. Further research is required to explore dietary acceptability, cost effectiveness and clinical effectiveness, prior to implementation alongside the current standard of care in this population.

Key areas for future research include:

- A pragmatic feasibility study to inform future RCT design; with outcomes related to adverse events, retention rates, quality of life, dietary acceptability, tolerability and compliance, using validated measures, as adopted by the KEATING study [32].
- Determining if KDs are effective in the management of glioma, through high quality RCTs. It will be important to consider which KD, if any, is beneficial and at what point in the treatment pathway. KD concurrent to chemoradiotherapy in animal models has proven to potentiate the treatment effects; however, this is yet to be replicated within glioblastoma patients. Testing clinical effectiveness for median OS, in a newly diagnosed glioblastoma population, would require approximately 600 participants and is therefore unlikely to be achieved. It may be practical to power studies to PFS or OS at 6 months to enable attainable recruitment figures.
- A health economic assessment to establish efficiency, clinical effectiveness and value of the intervention, would be beneficial. Establishing quality adjusted life years would be of benefit to assess disease burden, in terms of quality and quantity of life gained by patients, if at all any.

## Conclusion

This review is based on case series evidence, the lowest position in the evidence hierarchy. However, at present this is the only evidence available to inform decisions regarding the implementation of KDs for gliomas. While the review has found minimal adverse events, suggesting KDs to be safe in this population, the evidence for effectiveness and acceptability of various KDs is insufficient to suggest they have a therapeutic effect in the management of gliomas. Further high-quality research would be of benefit.

## Future perspective

Over the next 5 to 10 years we may see the field of KDs in gliomas expanding, following the publication of current ongoing studies. There will be the opportunity for meta-analysis presenting more robust evidence on the subject. However, it may be several years before large scale, RCT evidence is published exploring the effectiveness of KDs in gliomas.
