# Supplementary material for: The role of ketogenic diets in the therapeutic management of adult and paediatric gliomas: a systematic review
Source: CNS Oncol. 2018 Apr 16;7(2):CNS17. doi: 10.2217/cns-2017-0030 (PMC5977276; doi:10.2217/cns-2017-0030)
Supplement: Supplementary file 1 [file cns-07-17-s1.docx]

**Appendix 1**: **Example Search Strategy – Medline (Ovid)**

1. (ketogenic or ketone or ketosis or carbohydrate restrict* or low carbohydrate or high fat or Atkin* or glyc*emic or triglyceride* or medium chain triglyceride*).af
2. (central nervous system or brain or cerebral or spinal or spine).af
3. (cancer or tumo*r or malignan* or neoplas* or carcinoma*).af
4. 2 and 3
5. (glioblastoma* or astrocytoma* or glioma* or ependymoma* or oligodendroglioma* or ganglioglioma* or medulloblastoma* or astrocytic* or ependymal*).af
6. 4 or 5
7. 1 and 6
8. Limit 7 to (English language and humans)
